# Shining a light on the impact of antifungals on *Aspergillus fumigatus* subcellular dynamics through fluorescence imaging

**DOI:** 10.1128/aac.00803-24

**Published:** 2024-10-15

**Authors:** I. S. R. Storer, L. E. Sastré-Velásquez, T. Easter, B. Mertens, A. Dallemulle, M. Bottery, R. Tank, M. Offterdinger, M. J. Bromley, N. van Rhijn, F. Gsaller

**Affiliations:** 1 Manchester Fungal Infection Group, Division of Infection, Immunity, and Respiratory Medicine, University of Manchester, Manchester, United Kingdom; 2 Institute of Molecular Biology, Biocenter, Medical University of Innsbruck, Innsbruck, Austria; 3 Institute of Neurobiochemistry, Biocenter, Medical University of Innsbruck, Innsbruck, Austria; 4 Microbial Evolution Research Manchester, University of Manchester, Manchester, United Kingdom; University Children’s Hospital Münster, Münster, Germany

**Keywords:** *Aspergillus*, fluorescence, antifungal agents, fluorophores, *Aspergillus fumigatus*, fungal disease, imaging, microscopy

## Abstract

Fluorescent proteins (FPs) are indispensable tools used for molecular imaging, single-cell dynamics, imaging in infection models, and more. However, next-generation FPs have yet to be characterized in *Aspergillus*. Here, we characterize 18 FPs in the pathogenic filamentous fungus *Aspergillus fumigatus* spanning the visible light spectrum. We report on *in vivo* FP brightness in hyphal and spore morphotypes and show how a fluoropyrimidine-based selection system can be used to iteratively introduce four distinct FPs enabling the simultaneous visualization of the cell membrane, mitochondria, peroxisomes, and vacuoles. Using this strain, we describe and compare the dynamic responses of organelles to stresses induced by voriconazole, amphotericin B, and the novel antifungal drugs olorofim and manogepix. The expansion to the fluorescent genetic toolbox will overcome boundaries in research applications that involve fluorescence imaging in filamentous fungi.

## INTRODUCTION

*Aspergillus fumigatus* is a saprotrophic fungus found in a wide range of ecological niches, which can cause allergic, invasive, and chronic diseases in humans that are difficult to diagnose and treat (1). Recent estimates indicate that between one and two million people are diagnosed with life-threatening invasive aspergillosis annually, where mortality rates can exceed 40% (2–4). The first-line treatment for invasive infections is the triazoles; however, resistance to this class of compounds is increasing globally and is associated with poorer treatment outcomes [25% increase in mortality (2)]. Major gaps remain in our understanding of *A. fumigatus* pathobiology, virulence, treatment and evolution.

Molecular research on filamentous fungi such as *A. fumigatus* relies on the development of novel genetic tools and techniques, which have been mainly adapted from model organisms such as *Aspergillus nidulans* and *Neurospora crassa* (5, 6). In the past 20 years, multiple fluorescent proteins (FPs) have been developed that can be used in filamentous fungi, with sGFP, a synthetic GFP variant comprising a serine to threonine substitution at position 65 of the protein sequence, being the first one specifically designed for this task (7). Since then, in addition to GFP S65T, other fluorophores have been used in *A. fumigatus* covering blue, green, yellow, orange, and red FPs (8–15). These have enabled investigations into the developmental biology of this pathogen as well as the subcellular dynamic in response to specific treatments, including antifungals (16–18). These FPs originate from different sources; namely *Aequorea victoria* and *Discosoma* species and have different dimerization properties and different predicted brightness. This can lead to complications when visualizing proteins that are expressed at low levels, often leading to a need to overexpress the proteins of interest, which may not be physiologically relevant.

Significant advances in FP engineering have led to increased brightness and photostability, together with reduced oligomerization and maturation times (19–21). Photostability is a major consideration in live-cell fluorescence microscopy. Photobleaching measurements *in vivo* have been found to be consistent with measurements *in vitro*, but stability can be affected by the culture medium or microscopy technique: laser-scanning confocal microscopy, used in this work, bleaches FPs faster than widefield microscopy (22, 23). However, many FPs perform differently across different organisms. While many FPs have been developed for and evaluated in other fungal species such as *Candida albicans* (24), *Cryptococcus neoformans* (25) *N. crassa* (26), and *Saccharomyces cerevisiae* (27, 28)*,* no extensive direct comparisons of FPs have been carried out in *A. fumigatus*. Furthermore, characterizing a diverse set of FPs covering a wide range of wavelengths allows for multicolor imaging experiments and the potential to design Förster resonance energy transfer (FRET) systems (29, 30).

In this work, we characterize the brightness of cytoplasmic FPs that are expressed using a common *A. nidulans* promoter, *PgpdA* (31), from single-copy integrations at a defined locus within an isogenic strain of *A. fumigatus*. We describe a strain labelled with four different FPs, which was generated using four endogenous counter-selectable markers, enabling the simultaneous visualization of the mitochondria, peroxisomes, vacuoles, and the cell membrane. We use this strain to monitor the effects of voriconazole, amphotericin B, olorofim, and manogepix on these subcellular compartments at the population level. Finally, we examine the temporal response to manogepix, a first-in-class glycosylphosphatidylinositol biosynthesis inhibitor (32) at the individual level.

## MATERIALS AND METHODS

### Oligonucleotides, strains, and growth conditions

Oligonucleotides and plasmids used in this study are listed in Table S1. To generate conidia for experiments, strains were grown on solid *Aspergillus* minimal medium (AMM) (33) at 37°C for 5 days in vented tissue culture flask (Corning) and harvested through Miracloth (Millipore) in PBS + 0.01% Tween-20. Phenotyping on solid medium was carried out by point inoculation of 1 × 10^4^ of each strain in a total volume of 5 µL PBS + 0.01% Tween-20 on solid AMM, pH 5. Plates were incubated for 48 h at 37°C. Liquid growth curves were carried out by inoculating 5 µL of a 1 × 10^5^ spores/mL solution of each strain in 200 µL liquid AMM, pH 7. Microdilution plates were incubated for 48 h at 37°C, and optical density at 600nm (OD_600_) was measured every 10 min on a BioTek Synergy2 plate reader. Growth rates were calculated as the slope of the linear part of the growth curve (12–18 h). These were compared by a one-way ANOVA (*n* = 3) with Šidák multiple comparisons. *PgpdA*-FP-expressing strains were imaged in liquid AMM, pH 7. Germlings were grown by inoculating 1 × 10^4^ spores in 200 µL in a µ-Slide 8-well high glass bottom chamber (Ibidi) or a microfluidic chamber made in-house (described in section 2.3) at 30°C for 16 h. *PxylP*-FP-expressing strains were imaged in liquid RPMI-1640 supplemented with 1 mg/L amphotericin B (Sigma-Aldrich), 0.5 mg/L voriconazole (Sigma-Aldrich), 0.016 mg/L olorofim (Concept Life Sciences) or 0.6 mg/L manogepix (Selleck Chemicals), with 1% xylose to induce gene expression.

### Transformation and CRISPR-Cas9-mediated strain generation

FP knock-in cassettes were generated using fusion PCR (34) (Fig. S1a), or by linearization of plasmids where the *PxylP* promoter and FP were already sequential on a vector (see Table S1 and supplemental methods). The construction of plasmids and the tetrachrome strain is described in detail in the supplemental methods. Transformations were either performed by homologous recombination following Zhao *et al.* (35) or by CRISPR-Cas9-mediated transformation based upon (33). The method used to generate each strain is found in Table S2. In summary, *A. fumigatus* A1160P+ (36) was grown overnight at 37°C in a shake flask culture (130 rpm, 37°C). A protoplasting solution was made by dissolving 1 g/10 mL Vinotaste Pro (Novozymes) in 0.6 M KCl + 100 mM citric acid and added to the shake flask culture for 3 h at 130 rpm 37°C. Protoplasts were filtered through Miracloth to remove hyphae and debris, followed by three washes in 0.6 M KCl. Protoplasts were resuspended in 0.6 M KCl + 50 mM CaCl_2_. 25 µL of PEG6000 solution, and ~500 ng knock-in cassette was added to the protoplast suspension. If CRISPR-Cas9-mediated transformation was used, ribonucleoproteins (RNPs) were assembled by incubating crRNA with tracrRNA (Integrated DNA Technologies, Inc.) in duplex buffer and further incubated with purified SpCas9 (Integrated DNA Technologies, Inc.). RNPs were added at this stage. Protoplast suspensions were incubated for 50 min on ice. 600 µL of PEG6000 solution was added followed by incubation for 20 min at room temperature. Suspensions were plated onto AMM + 1 M sucrose-containing 100 mM citrate buffer (pH 5). Selection procedures using counter-selectable markers *fcyB*, *fcyA*, and *uprt* were conducted as described previously for *A. fumigatus* (14, 37, 38). In the case of *cntA*-based counter-selection, plates containing 50 µg/mL 5-fluorouridine (5FUR) were supplemented with 50 µg/mL of clorgyline (CLG). Strain descriptions and sources for each FP used in each cassette are found in Tables S2 and S3, respectively.

### Fluorescence microscopy image acquisition

**FIG 1:**
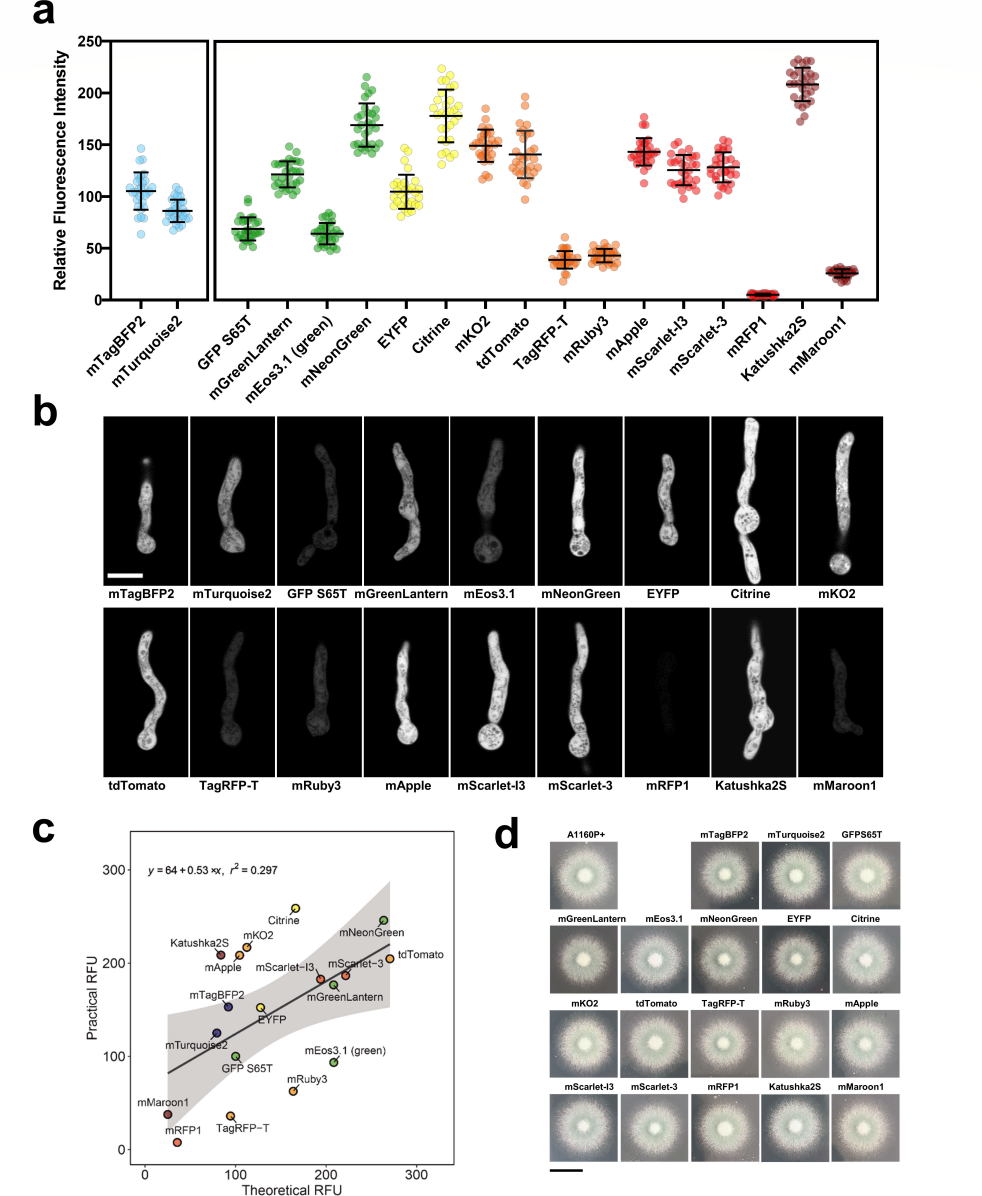
Brightness of 18 FPs in *A. fumigatus* hyphae using confocal microscopy. (a) Strains expressing each FP were imaged via confocal microscopy. Fluorophores were excited at the peak excitation derived from FPbase and captured at the peak emission ±10 nm bandwidth. mTagBFP2 and mTurquoise2 were excited at 405 nm due to white light laser constraints. Thirty germlings were assessed via ImageJ for each strain. Lines are at mean ± SD. (b) Cytosolic expression of FPs within *A. fumigatus* germlings. Representative images were captured via confocal laser scanning microscopy using a 63× objective. Germlings were grown in AMM for 18 h at 30°C and 1 h at 37°C. Scale bar, 5 µm. (c) Practical vs. theoretical brightness of FPs in *A. fumigatus* germlings. Theoretical brightness values were extracted from FPbase (molecular brightness), relative to the molecular brightness of GFP S65T. Practical brightness values were extracted from mean values from the brightness in *A. fumigatus* germlings. Theoretical and practical brightness values were moderately positively correlated [Pearson’s correlation Theoretical: Practical *T*_17_ = 2.7877, *P* = 0.01263, *r* (17) = 0.314]. Shaded areas show a 95% confidence interval. (d) Plate growth assays of FP-expression strains. Strains were point inoculated on solid AMM pH 5, and images were acquired after 48 h of incubation at 37°C. Scale bar, 1 cm.

The microfluidic device utilized in this study is fabricated through a process involving photolithography and dry etching to create a negative template. Polydimethylsiloxane (PDMS) is then cast using a 1:5 ratio based on the layout defined by the negative. Inlets and outlets are created by punching holes into the PDMS. Following this, the PDMS and KOH-cleaned glass substrate are subjected to plasma treatment to enhance bonding. The glass substrate and the channel side of the PDMS are then brought together, forming a chemically bonded structure. To reinforce the bonding, the assembled device is placed glass side down on a hotplate at 90°C for 10 min. After cooling, the channels are cleared by injecting a 1:1,000 Tween-20 dilution for 30 min, followed by inoculation with cell suspensions. The spores are allowed to incubate for an hour before applying a flow rate of 0.8 mL/h through the channels. Germlings were grown as in section 2.1. To confirm the predicted vacuolar localization of Vam3 along the hyphae, Δ*fcyA::GFP S65T-Vam3^PxylP^* cultures were stained with 2 µM of CellTracker Blue CMAC dye and further incubated for about 45–60 min as previously described for *A. fumigatus* (17). Fluorescence microscopy images were captured with a fully motorized Leica SP8x laser scanning confocal microscope equipped with a 40×/0.85NA HCX PL APO dry objective or a 63×/1.4NA HC PL APO CS2 oil objective. Imaging was performed at 37°C. All images were captured in 8-bit at 1,040 × 1,040 pixels. Blue FPs were excited using the 405 nm diode laser at 10%. All other FPs were excited using a white light laser at 20%. Excitation wavelengths were chosen to match the excitation maxima of the FPs, apart from mTagBFP2 and mTurquoise2 where the 405 nm diode laser was used. The fluorescence signal was captured in a 20 nm bandwidth spanning the maximum emission. For relative brightness measurements (Fig. 1a), the 40× objective with a pinhole of 1 AIRY unit, a scan speed of 400 Hz, and a line average of 4 was used. The gain was set to 300%. For the representative image (Fig. 1b), the 63× objective with 3× zoom, a pinhole of 1 AIRY unit, a scan speed of 400 Hz, and a line average of 8 was used. The gain was set to 150%. FPs were captured using HyD detectors. For the tetrachrome strain, mTagBFP2^per^ was excited at 405 nm with a diode laser. The remaining FPs were excited with at white light laser at the following wavelengths GFP S65T^vac^ at 490 nm captured, Katushka2S^mit^ at 670 nm, and mKO2^mem^ at 588 nm.

### Quantitative measurement of relative fluorescent signal

Thirty individual germlings from each strain were assessed for FP relative fluorescent signal. The mean grey value of 30 2 × 2 µm sections deemed in focus by viewing the brightfield image was quantified. Fluorescence intensities are displayed in arbitrary units. For each strain, the same settings (magnification, scan speed, laser power, emission range, gain) were used on the background strain, A1160P+, to capture autofluorescence at each excitation/emission. This value was subtracted to give the practical brightness value. Relative fluorescent intensity plots were processed using GraphPad Prism 9.3.1. The brightness of mTagBFP2 and mTurquoise2 were compared using an unpaired *t*-test. The brightness of all other FPs were compared using a Kruskal–Wallis test with Dunn’s multiple comparisons.

### Imaging flow cytometry

Fluorescent strains were individually cultured on Sabouraud dextrose (SAB) agar for 72 h at 37°C. Spores were harvested with spore buffer for imaging flow cytometry processing. For each strain, 5,000 events were recorded with two technical replicates. All images and data were collected using an ImageStreamX Mark II Imaging Flow Cytometer (Merck). Total events were filtered to exclude speed beads, clumps of spores and out-of-focus spores. Fluorescence data were collected using IDEAS software. Excess speed bead images acquired were further excluded through gating. Gating to distinguish between spores and beads in scatter channel was determined by locating the area of spores using previously used fluorophore tdTomato in channel 3 (560–595) and applying the region containing all spores onto the brightfield channel. Median relative fluorescence intensity values were collected for each fluorophore across six channels: (Channel 1 – excitation 488 nm (emission 480−560 nm), Channel 2 – excitation 488/561 nm (emission 560−595 nm), Channel 3 – excitation 488/561 nm (emission 595−640 nm), Channel 4 – excitation 488/561 nm (emission 640−745 nm), Channel 5 – excitation 405 nm (emission 430−505 nm), Channel 6 – excitation 642 nm (emission 640−745 nm) . All fluorescence values were compared relatively to the non-fluorescent strain A1160P+. Fluorescence density plots were created in R using the ggplot2 package. The interacting effects of practical brightness and theoretical brightness were calculated using simple linear regression.

### Quantitative measurement of drug effects on the tetrachrome strain

Data were acquired using ImageJ. Distance between neighboring peroxisomes was measured by auto-thresholding the image and using the nearest neighbor plugin (author Yuxiong Mao). Vacuole area was measured using the “analyze particles” function with a minimum size of five pixel units and a circularity of 0.20–1.00. Mitochondrial fragmentation was measured using the “analyze particles” function with a minimum size of 10 pixel units. Plots and statistical analyses were processed using GraphPad Prism 9.3.1. Significance was determined using a Kruskal–Wallis test with Dunn’s corrects for vacuole area, peroxisome internal, and mitochondrial fragmentation. To model the effect of exposure time of DSMO on mitochondria and vacuole area a mixed effects linear model was fitted to the data using the lmer package in R. The model fit the mitochondria area or vacuole area to the interacting effects of time and DSMO treatment, with replication modelled as a random effect. The model allowed the intercept and the slope to differ between treatments and replicates which resulted in a lower Akaike’s Information Criterion. Normality and linearity of the residuals satisfied the assumptions of the model.

## RESULTS

### Generation of a brighter FP palette in *A. fumigatus*

Fluorescence imaging in *A. fumigatus* has relied mostly on a limited number of fluorophores, with the avGFP derivatives; even though GFP, eGFP, and GFP S65T are most commonly used (39), however, the relatively low brightness of these FPs makes visualizing proteins that are expressed at low levels difficult. Paired with stability and oligomerization issues (40, 41), this makes the current FP palette sub-optimal in *A. fumigatus*. However, some next-generation FPs are documented to be brighter, have more compatible spectra for multicolor imaging, and tend to be monomeric, overcoming many of the previous caveats for using FPs in *A. fumigatus*. The use of such FPs opens up novel applications such as single-cell experimentation. To assess next-generation FPs in *A. fumigatus,* we gathered a collection of fluorophores that have been used in *C. neoformans*, *N. crassa* and several fluorophores that have shown excellent performance in mammalian cells: mTagBFP2 and mTurquoise2 (blue); GFP S65T, mGreenLantern, the photoswitchable mEOS3.1, and mNeonGreen (green); EYFP and Citrine (yellow); mKO2, tdTomato, and TagRFP-T, and mRuby3 (orange); mApple, mScarlet-I3, mScarlet-3, and mRFP1 (red); and Katushka2S and mMaroon1 (far-red). These fluorophores span the visible spectrum, have different excitation optima, and have different oligomerization states (Table S3).

To enable systematic testing of the relative brightness of each FP, we generated strains expressing each FP driven by the *A. nidulans gpdA* promoter from the *fcyB* locus (Fig. S1a) (14, 42) as a single-copy integration (Fig. S1b). To assess the practical brightness of this set of FPs in *A. fumigatus* germlings, we imaged each strain and measured the relative fluorescence intensity using confocal microscopy (*n* = 30) (Fig. 1a). To compare FPs, the peak excitation wavelength was used and a 20 nm bandwidth spanning the maximum theoretical emission wavelength. However, it should be noted that excitation at 405 nm was used for mTurquoise2 and mTagBFP2 due to laser constraints and were therefore analyzed separately. The mean practical brightness ± SD for each FP is shown in Table 1. In the blue channel, the brightest blue protein was mTagBFP2, which was 22% brighter than mTurquoise2 (*t* = 4.999, df = 58, *P* < 0.0001). The brightest green protein was mNeonGreen, which was 146% brighter than GFP S65T (*P* < 0.0001). The yellow fluorescing protein Citrine was 159%, which was brighter than GFP S65T (*P* < 0.0001) and 70% brighter than EYFP (*P* < 0.0001). The brightest orange FP, mKO2, was 117% brighter than GFP S65T (*P* < 0.0001) and 283% brighter than the least bright orange FP, TagRFP-T (*P* < 0.0001). Aggregates could be observed in mKO2-expressing hyphae, apparent as bright areas ~0.5 µm in diameter (Fig. 1b), suggesting that this protein is poorly tolerated when overexpressed in the cytoplasm under constitutive expression in *A. fumigatus*, similar to previously reported results in *S. cerevisiae* (28). mApple was the brightest red FP, which was 108% brighter than GFP S65T (*P* < 0.0001) and 2,638% brighter than the least bright red FP, mRFP1 (*P* < 0.0001). While mRFP1 was considered the least bright fluorophore in all tested strains, we could still observe fluorophore under the microscope upon increasing the exposure time and gain. Katushka2S was the brightest overall FP in hyphae. It was 203% brighter than GFP S65T (*P* < 0.0001) and 705% brighter than the other far-red FP tested here, mMaroon1 (*P* < 0.0001).

**TABLE 1:** Relative brightness of FPs used in this work^*a*^^,*b*^

FP	TB	TB relative to GFP S65T (%)	Relative brightness in hyphae	Relative brightness in spores
			Mean PB ± SD	Median PB ± SD
mTagBFP2	32.38	91.99	105.25 ± 18.05	12,755.9 ± 18,065.9
mTurquoise2	27.90	79.26	86.04 ± 10.81	4,909.4 ± 2,139.7
GFP S65T	35.20	100.00	68.84 ± 11.11	27,042.8 ± 11,988.3
mGreenLantern	73.30	208.24	121.60 ± 12.53	36,189.8 ± 16,735.6
mEos3.1 (green state)	73.37	208.44	64.30 ± 10.32	48,706.6 ± 20,583.1
mNeonGreen	92.80	263.64	169.26 ± 20.90	19,247.1 ± 10,058.1
EYFP	44.89	127.53	104.83 ± 16.46	492.9 ± 8,022.2
Citrine	58.52	166.25	178.12 ± 25.45	36,709.4 ± 15,740.8
mKO2	39.56	112.39	149.27 ± 15.62	84,972.1 ± 33,642.4
tdTomato	95.22	270.51	140.85 ± 22.93	48,122.2 ± 25,771.4
TagRFP-T	33.21	94.35	38.96 ± 8.48	2,154.1 ± 1,793.5
mRuby3	57.60	163.64	43.04 ± 6.52	5,467.1 ± 2,452.0
mApple	36.75	104.40	143.45 ± 13.20	2,660.5 ± 1,601.1
mScarlet-I3	68.25	193.89	125.77 ± 14.65	7,729.53125 ± 3,345.3
mScarlet-3	78.00	221.59	128.41 ± 14.44	16,116.7 ± 6,162.5
mRFP1	12.50	35.51	5.24 ± 1.06	8,277.2 ± 3,618.6
Katushka2S	29.48	83.75	208.60 ± 16.17	15,794.9 ± 9,506.7
mMaroon1	8.80	25.00	25.92 ± 3.96	736.3 ± 488.0

^*a*^ TB data are from FPbase (https://www.fpbase.org). PB is calculated in this work.

^*b*^ PB, practical brightness; TB, theoretical brightness.

A moderate but significant positive correlation was found between the mean practical brightness of each FP compared to the theoretical brightness from FPbase [Pearson’s correlation Theoretical: Practical *T*_17_ = 2.7877, *P* = 0.01263, *r* (17) = 0.314] (Fig. 1c). FPs such as mRuby3 or mEOS3.1 (in the un-photoconverted, green state) are brighter in theory—when calculated as the product of extinction coefficient and quantum yield (43)— compared to how they perform in *A. fumigatus*, whereas Citrine and KatushkaS2 appear brighter *in vivo*. As *PgpdA* is reported to induce high expression (44, 45), we assessed the potential growth defects of strains expressing FPs. *PgpdA-driven* overexpression caused no growth defects in solid media (Fig. 1d) or had a significant effect on the growth rate in liquid media (Fig. S1c).

As many experimental setups in *A. fumigatus* rely on the infectious propagules, conidia, we assessed fluorescent signals of all the proteins when expressed in conidia. Conidia produced by all fluorescent constructs were distinguishable from non-fluorescent wild-type spores in at least one channel (Fig. S2). The brightest FP in the 430–505 nm channel was mTurquoise2. The brightest in the 480–560 nm channel was mEOS3.1. In the 560–595 nm channel, mKO2 was the brightest FP. Only a single FP showed peak emission within the 595–640 nm channel (mScarlet-3) and in the 640–745 nm channel (Katushka2S).

### Multi-organelle imaging using the tetrachrome *A. fumigatus* allows tracking of the response to antifungals

We have previously described a strategy to integrate three DNA sequences of interest into the *A. fumigatus* genome by exploiting the pyrimidine salvage pathway (14). In *A. nidulans*, CntA (NCBI accession number: XP_663097, *A. fumigatus* homologue AFUB_001570) has been described as the main importer of 5FUR (46). As the activity of 5FUR can be enhanced by the addition of the broad-spectrum inhibitor of fungal efflux pumps CLG (47, 48), we used a combination of 50 µg/mL of 5FUR with 50 µg/mL CLG to improve selection for *cntA*-disrupted colonies (Fig. S3a). This way, we were able to use *cntA* together with the endogenous counter-selectable markers *fcyB*, *fcyA* and *uprt* in a sequential manner (Fig. S3b and c). Replacement of all four loci did not affect *A. fumigatus* growth morphology on a plate assay (Fig. S3d). In addition, the replacement of all four loci did not change the MIC/MEC to voriconazole, amphotericin B, olorofim, and manogepix compared to the wild-type strain. Also, addition of xylose to the culture medium did not change the MIC/MEC to these antifungals (Fig. S4f). Constructs that were integrated at these genomic loci were used to visualize the mitochondria using the CitA_40_ sequence (49), vacuoles by tagging the *Aspergillus oryzae* Vam3 homologue (50), the peroxisomes using PTS1-consensus tripeptide SKL (51), and the cell membrane tagging the *A. nidulans* homologue UapC (52). This single strain, referred to as the tetrachrome strain, allows the visualization of mitochondria (CitA_40_-Katushka2S), vacuoles (GFP S65T-Vam3), peroxisomes (mTagBFP2-SKL) and cell membrane (UapC-mKO2) simultaneously. We further assessed the potential effect of ectopic expression of GFP S65T tagged Vam3 by simultaneously staining with CMAC, which showed colocalization to the vacuoles as normal (Fig. S3f). Signal from FP expression under the control of the xylose inducible promoter was quantifiable after 1 h and signal increased during the observation period (Fig. S3g). Growth of the tetrachrome strain did not differ from the wild-type strain in different concentrations of xylose (Fig. S3h).

**FIG 2:**
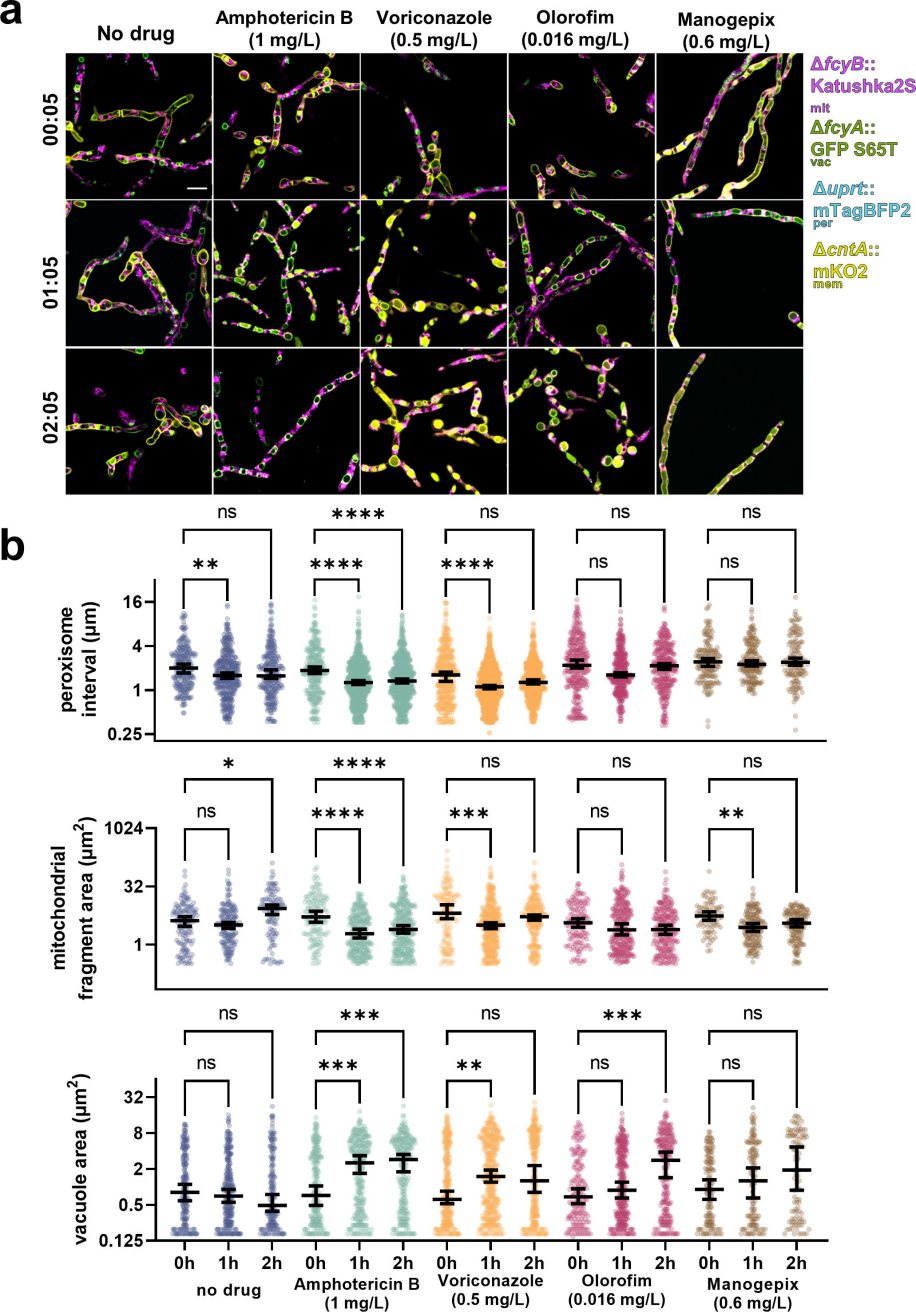
Multi-organelle tracking of the effect of antifungals using four FPs. (a) *A. fumigatus* hyphae were grown in RPMI-1640. Representative images of the four-color strain imaged in RPMI-1640 with no drug, 1 mg/L amphotericin B, 0.5 mg/L voriconazole, 0.016 mg/L olorofim, or 0.6 mg/L manogepix. Images were acquired 5 min after the addition of the drug, and 1 and 2 h post-addition of the drug. Scale bar, 10 µm. (b) Quantification of the distance between neighboring peroxisomes, mitochondria fragment area, and vacuole area for each condition. Lines are at medians with a 95% confidence interval. Significance was determined using a Kruskal–Wallis test with Dunn’s corrections (*<0.05, **<0.01, ***<0.001, ****<0.0001).

To investigate subcellular dynamics in the tetrachrome strain over a period of 2 h (Fig. 2a), we assessed the four tagged organelles simultaneously using automated analysis (Fig. 2b, individual channels in Fig. S4). The distance between peroxisomes (peroxisome interval) was measured as a proxy for peroxisome abundance to account for growing hyphae. This showed that within the first hour, the peroxisome interval became smaller (0 h median 2.002 µm, 1 h median 1.583 µm) indicating that as hyphae age, peroxisomes become more abundant (*P* = 0.0059). Within the first 2 h, we observed that mitochondrial fragments became larger (0 h median 4.197 µm, 2 h median 8.622 µm, *P* = 0.04), which was likely due to cellular growth. The vacuole area remained unchanged.

Next, we investigated the effect of antifungals on the tetrachrome strain. We exposed hyphae grown for 18 h to 2× MIC amphotericin B and monitored the effects on organelles. Amphotericin B exposure significantly reduced the peroxisome interval over 1 h (0 h median 1.615 µm, 1 h median 1.108 µm, *P* < 0.0001) and 2 h (2 h median 1.276 µm, *P* < 0.0001). This indicates more peroxisomes per cellular volume. Compared to the wild-type strain, the peroxisomes differed after 1 and 2 h (both *P* < 0.0001). We observed the mitochondrial fragment size decreased over the course of 2 h (0 h median 5.206 µm, 2 h median 2.473 µm, *P* = 0.02), but this was not statistically different to the no-drug control (*P* = 0.87). The vacuole area increased over the same period (0 h median 0.618 µm, 1 h median 1.479 µm, *P* = 0.0014), which was significantly different to the no-drug control (*P* = 0.0006). Hyphae treated with amphotericin B for 2 h were visually deflated (Fig. 2a), suggestive of hyphal elements dying from exposure to the drug and organelle degradation.

We further investigated the effect of 2× MIC voriconazole on cellular morphology. An increase in vacuole area was observed (0 h median 0.72 µm, 2 h median 2.54 µm, *P* = 0.0004) after 1 h, which seemed to plateau at 2 h, seemingly distinct from the vacuole behavior seen for amphotericin B indicating an adaptive response to voriconazole includes compartmentalization of the drug, a toxic by-product resulting from drug action or the recycling of proteins. Additionally, a statistically significant difference to the no-drug control was observed for peroxisome interval after 1 (*P* < 0.0001) and 2 h (*P* = 0.0019), and for mitochondrial fragmentation at both time-points (1 h *P* = 0.01, 2 h *P* < 0.0001). Upon 2× MIC olorofim exposure, no statistical difference was observed in the peroxisome interval and mitochondrial fragment area. Vacuole area increased after 2 h upon olorofim exposure (0 h median 0.68 µm, 2 h median 2.79 µm, *P* = 0.0002). Increased vacuole size has previously been documented in response to olorofim exposure (17). Our data reveal a direct correlation between the increase in vacuole size and mitochondrial fragmentation following exposure to voriconazole and amphotericin B. It is not clear whether the apparent fragmentation is caused by a direct effect of the compounds on mitochondrial structure, or an indirect effect of the vacuoles spatially restricting mitochondrial structure. We also observed the colocalization of UapC-mKO2 with the vacuoles during voriconazole treatment. This colocalization also occurs with olorofim and manogepix treatment. This translocation of UapC from the plasma membrane to the vacuolar compartment has been observed in *A. nidulans* upon ammonium exposure (53). It is not clear what exactly is causing the translocation of UapC to the vacuoles.

### Temporal changes in organelles in response to manogepix exposure

**FIG 3:**
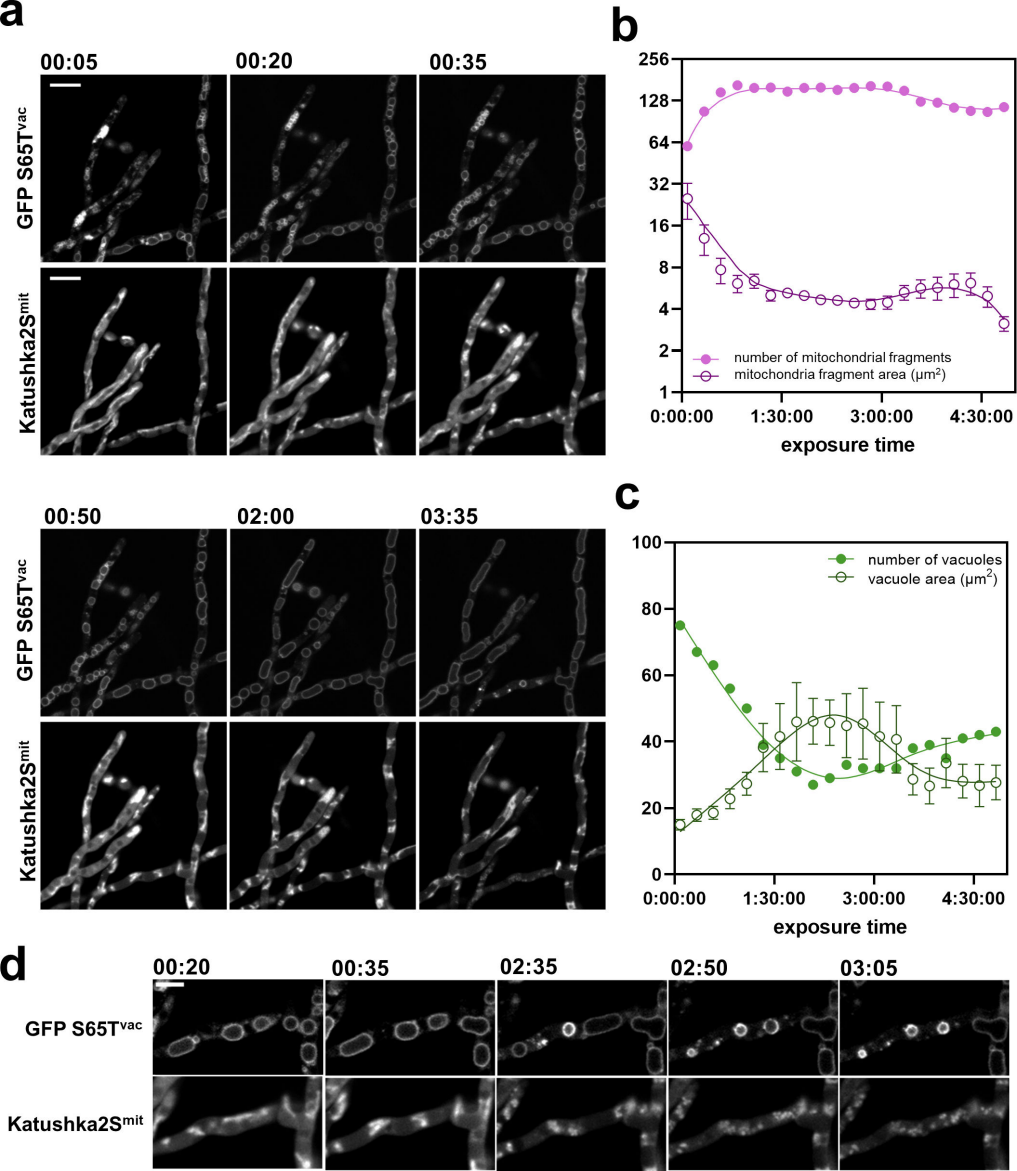
High-resolution organelle dynamics upon manogepix exposure. (a) *A. fumigatus* hyphae were grown in RPMI-1640 and imaged after the addition of 0.6 mg/L manogepix. Images of the mitochondria and the vacuoles were acquired 5 min after the addition of the drug, and then in 15 min intervals. Scale bar, 10 µm. Quantification of the (b) number and area of mitochondrial fragments, and (c) number and area of vacuoles, were performed using ImageJ. (d) Vacuole morphology during manogepix treatment. Scale bar, 5 µm.

Lastly, we investigated the effect of 2× MEC manogepix exposure on subcellular morphology. Surprisingly, no statistical difference in peroxisome interval, mitochondrial fragment area, and overall vacuole area was found. However, we saw a clear change in the distribution of vacuolar size. In the absence of drug, vacuoles exhibited a Gaussian distribution; however, upon the application of manogepix distribution of vacuole size was the binary response (large > 6 µm^2^ or small < 0.3 µm^2^, Fig. 2b). To investigate the response to manogepix in more detail, we looked at individual hyphae over 4.5 h at 15 min intervals (Fig. 3). The addition of drug vehicle, in this case DMSO, did not significantly increase or decrease vacuole or mitochondria fragment area over time (Fig. S5b and c). During manogepix exposure, mitochondrial fragments within individual hyphae became more numerous, and the mean mitochondrial fragment size decreased. At 5 min post-exposure, the mitochondria appeared tubular. There were 60 mitochondrial fragments, and the mean fragment area was 25.09 µm^2^ (±7.32 SEM). Fragmentation of the mitochondrial network was apparent by 20 min and most obvious by 35 min. At 35 min, the mean mitochondrial fragment size was 7.686 µm^2^ (±1.59 SEM), and the number of fragments increased to 147. After 35 min, the number of mitochondrial fragments plateaued (Fig. 3b). Over time, vacuoles appear to fuse. At 5 min manogepix exposure, there were 75 vacuoles, and the average area was 14.97 µm^2^ (±1.56 SEM) (Fig. 3c). At 2 h, vacuole size increased to an average of 46.15 µm^2^ (±6.91 SEM) and the number of vacuoles decreased to 27 (Fig. 3c). After 3 h, vacuole area appears to decrease (Fig. 3a, lower hyphae at 3.5 h). Interestingly, loss of signal is detected in these hyphae in the mitochondria (Katushka2s) channel by 3.5 h. We observed disruptions in the vacuoles, in which they suddenly burst and condensed, potentially releasing their contents (Fig. 3d).

## DISCUSSION

There are currently >1,000 entries of FPs on the open-source community database https://www.fpbase.org/ (27 August 2024). An ever-increasing choice of FPs with increased brightness, an increasing number of monomeric fluorophores, and longer lifetime properties are becoming more available. However, a well-performing fluorophore in one organism or experimental design may not translate to a different system (54). Therefore, we sought to explore a palette of next-generation FPs in *A. fumigatus*, the major mould pathogen of humans, to be used for multicolor imaging, allowing for temporal imaging of responses to antifungals.

First, we determined the *in vivo* brightness of 18 FPs in both hyphae and spores. The majority of FPs investigated have markedly improved brightness compared to the widely used fluorophore, GFP S65T. The brightest blue we found was mTagBFP2. The brightest green was mNeongreen, derived from lanYFP of *Branchiostoma lanceolatum* (55). This fluorophore has recently been used in *A. fumigatus* integrated into the genome to tag a protein, although under high expression levels (56). The next brightest green FP was mGreenlantern, derived from avGFP of *A. victoria*. Citrine and mTurquoise2 have also been derived from this FP, potentially highlighting that certain fluorophore lineages or sources might perform better than others in *A. fumigatus*. Similarly, mTagBFP2, Katushka2S, and mMaroon1 are all derived from eqFP578 of *Entacmaea quadricolor*. The brightest orange mKO2 is the only FP derived from KO of *Verrillofungia concinna*. While we did not attempt different codon optimization algorithms, in *C. albicans,* there is not one clear strategy to improve FP characteristics by codon optimization (24). Improvements to codon optimization may lead to even further improvements to FP performance.

These next-generation FPs can be used in microscopy to investigate cell biological phenomenons in general and aid antifungal drug discovery in particular, by identifying protein-protein interaction inhibitors, assessing drug effects in subcellular structures, or studying drug targets (17, 57–59). Using brighter FPs can be used at lower expressed proteins broadening their range of applications to include fluorescent genetic barcoding, gene expression reporters, and host-pathogen interactions. Overcoming oligomerization issues with FPs, using monomeric FPs can allow for more robust use of a split-FP system, overcome protein aggregation issues, and reduce background noise. In addition, we have characterized far-red shifted proteins in *A. fumigatus;* this may allow for deeper tissue imaging in infection models such as zebrafish, which have been previously described using *C. albicans* (60).

We used FPs with minimal spectral overlap to generate the tetrachrome strain to visualize the cell membrane, mitochondria, peroxisomes, and vacuoles simultaneously. Four-color imaging has previously been described in *N. crassa* (26), and *S. cerevisiae* (61), but to our knowledge, this is the first time, four FPs have been used simultaneously in a human pathogenic fungus. We expanded the genetic marker toolbox with the new endogenous counter-selectable marker *cntA*, to achieve in combination with the previously described markers *fcyB*, *fcyA*, and *uprt*, the insertion of four different FP-fusion cassettes. Importantly, the simultaneous loss of *fcyB*, *fcyA*, *uprt,* and *cntA* did not compromise *A. fumigatus* growth and development (Fig. S3d and h).

We investigated the changes in the subcellular compartments in response to two well-characterized antifungal drugs with different mechanisms of action: amphotericin B and voriconazole. Our data suggest a generalized response to both drugs, in which the distance between peroxisomes decreases, indicating an increase in peroxisomes. Peroxisomes are important in *A. fumigatus* to overcome oxidative stress (62), which has been known to be induced by the azoles and amphotericin B (63, 64). Mitochondria became fragmented in response to both antifungals. This switch from tubular to clustered and fragmented mitochondria has previously been observed in response to oxidative stress, cell death,and azole resistance (62). A sustained increase in vacuole size was only seen upon amphotericin B exposure. Vacuole disruption has been observed in response to amphotericin B in *C. albicans* (65) and *S. cerevisiae* (66). Our results highlight that the mechanism of action correlating with effects on subcellular structures could be elucidated via analysis of our tetrachrome strain.

We further characterized the response to the novel antifungals olorofim and manogepix, both of which are currently in the antifungal pipeline (67). In line with previous findings, we found vacuoles to become larger upon olorofim exposure (17). Upon manogepix exposure, we could not find any statistical differences in the morphology of peroxisomes, mitochondria, or vacuoles after 2 h of exposure. However, a clear biphasic response in the vacuole area was seen, which we explored in detail. We observe rapid mitochondrial fragmentation upon manogepix exposure, a known precursor for fungal cell death (62). Our data suggest that vacuoles rapidly enlarge and fuse in the first 2 h of exposure, and then diminish in size, possibly due to releasing their contents. However, the precise mechanisms driving these observations remain to be further investigated.

In summary, our results open new opportunities to advance fluorescence imaging in *A. fumigatus*. Our identification of successful FPs in *A. fumigatus* will provide valuable tools for not only molecular assays but also drug discovery and efficacy studies. We describe new approaches to evaluate and quantify the mechanisms behind antifungal treatments and reiterate the need for *in vivo* assays to validate FP usability in the species of choice as described in a previous work on FPs in other fungal species.

## SUPPLEMENTAL MATERIAL

Supplemental figures aac.00803-24-s0001.pdf Figures S1 to S5.

Supplemental methods aac.00803-24-s0002.docx Supplemental materials and methods on strain generation.

Supplemental tables aac.00803-24-s0003.xlsx Tables S1 to S3.

ASM does not own the copyrights to Supplemental Material that may be linked to, or accessed through, an article. The authors have granted ASM a non-exclusive, world-wide license to publish the Supplemental Material files. Please contact the corresponding author directly for reuse.
