# Supplementary material for: Shining a light on the impact of antifungals on Aspergillus fumigatus subcellular dynamics through fluorescence imaging
Source: Antimicrob Agents Chemother. 2024 Oct 15;68(11):e00803-24. doi: 10.1128/aac.00803-24 (PMC11539212; doi:10.1128/aac.00803-24)
Supplement: Supplemental figures — Figures S1 to S5. [file aac.00803-24-s0001.pdf]

S1

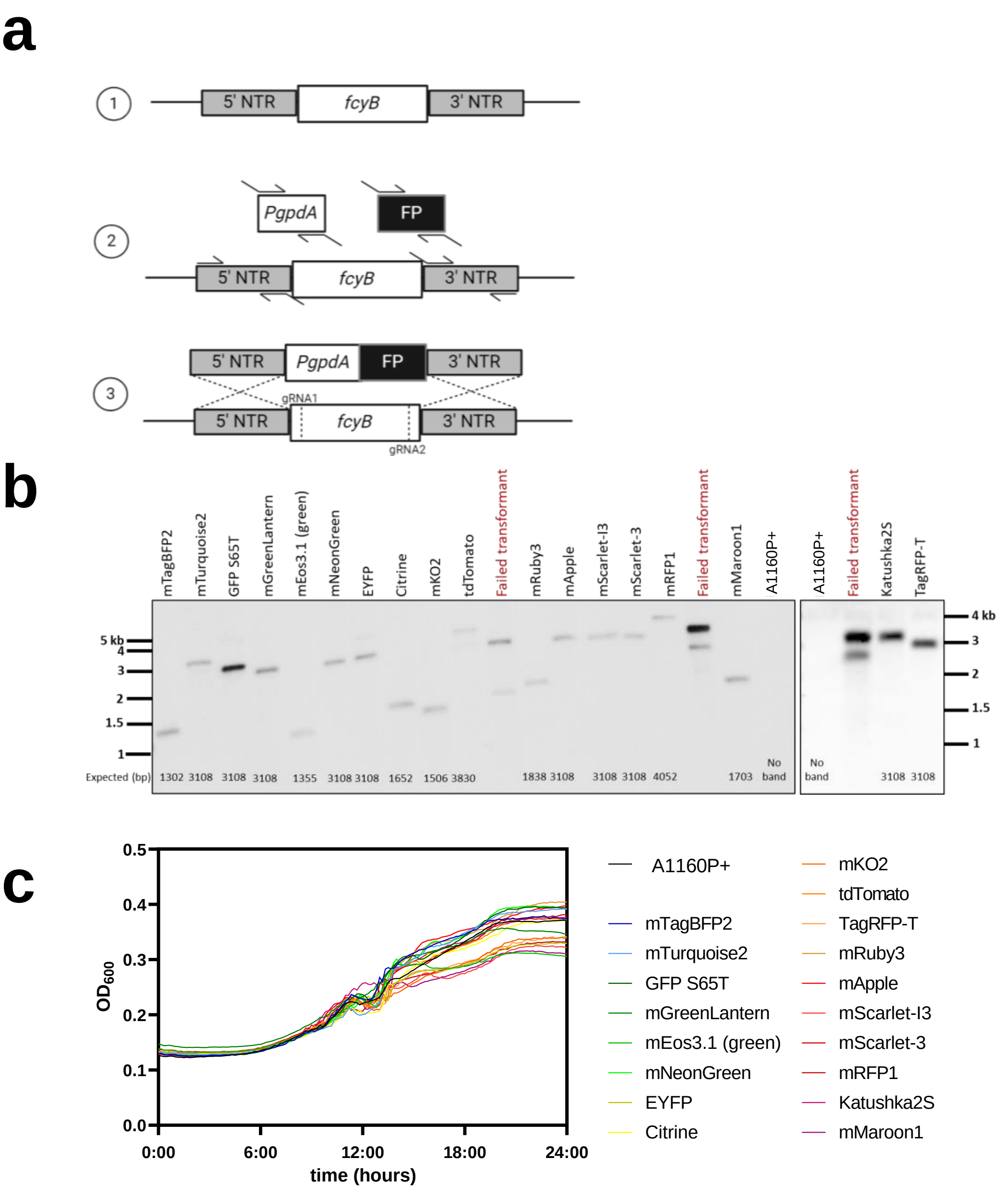

Figure S1. (a) Schematic of Fusion PCR mediated generation of knock-in constructs targeting the *fcyB* locus. The *PgpdA*, FP, and 5′ and 3′ nontranslated regions (NTRs) are amplified from plasmid DNA and genomic DNA. Both NTRs, *PgpdA*, and FP contain overlapping DNA (grey line) for subsequent connection via fusion PCR, yielding the knock-in constructs. Either HDR-mediated or CRISPR-Cas9-mediated (using gRNAs targeted dotted line regions) facilitate transformation. (b) Southern blot analysis for single copy FP cassette integrations. Expected band sizes are indicated. (c) Liquid growth rates of *PgpdA*-driven FP constructs. 5 µL of a 1 x 10^5 spores/mL solution of each strain were inoculated in liquid AMM, pH 7. Microdilution plates were incubated for 24 h at 37 °C with OD_{600} measurements captured every 10 minutes. Growth rates were calculated as the slope of growth between 12-18 hours. These were compared by a one-way ANOVA.

S2

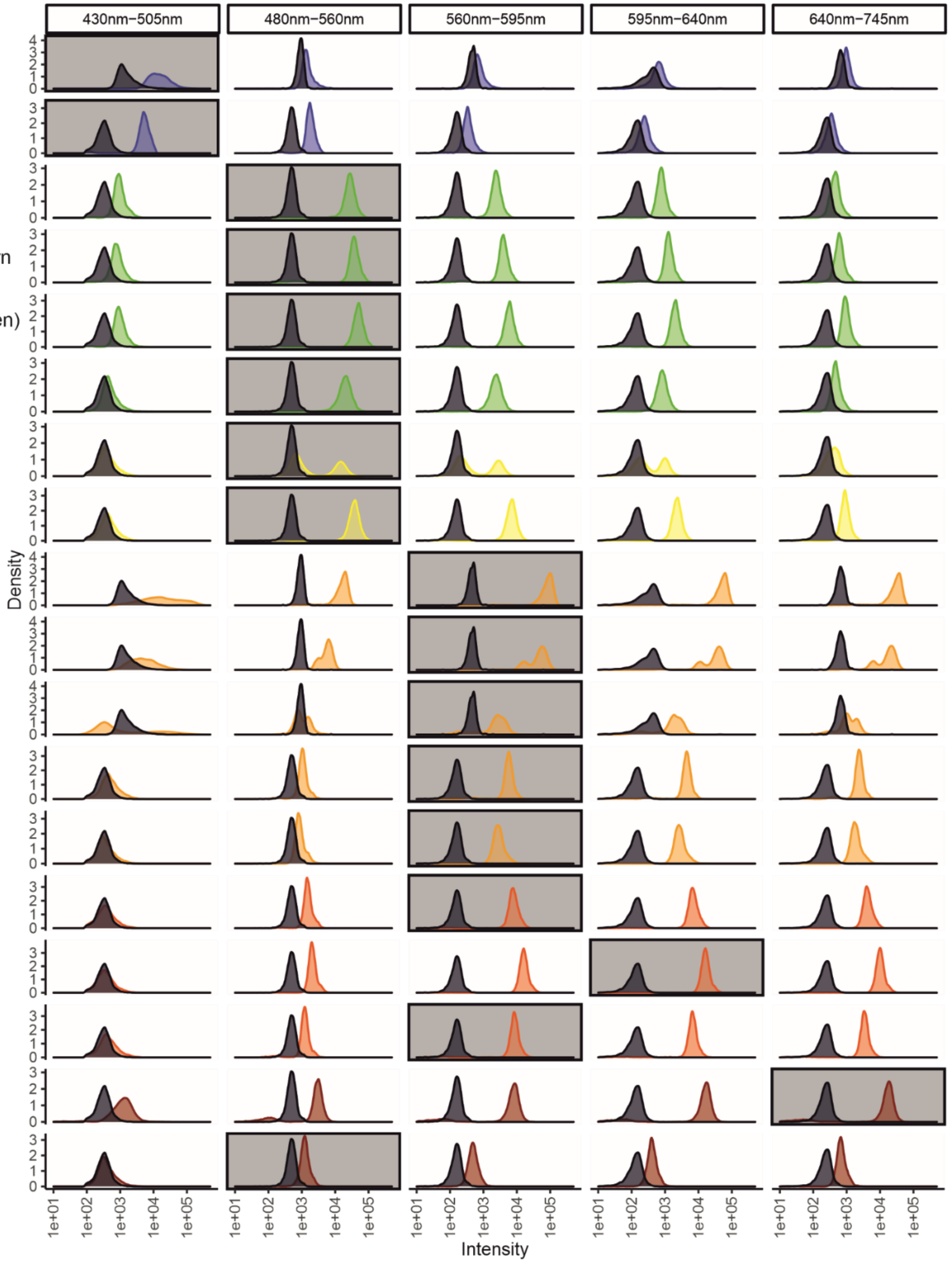

Figure S2. Brightness of 18 fluorescent proteins in spores using imaging flow cytometry. Population density plots faceted by an emission bandpass filter and fluorescent protein. Densities shown in grey represent wild-type A1160P+ (WT) population emissions. Strains expressing mTagBFP2, mKO2, tdTomato and TagRFP-T were conducted in a separate experiment with separate internal WT control. Density plots with the greatest difference between the median fluorescent intensity of the WT strain and fluorophore-expressing strain are highlighted for each fluorescent protein.#

S3

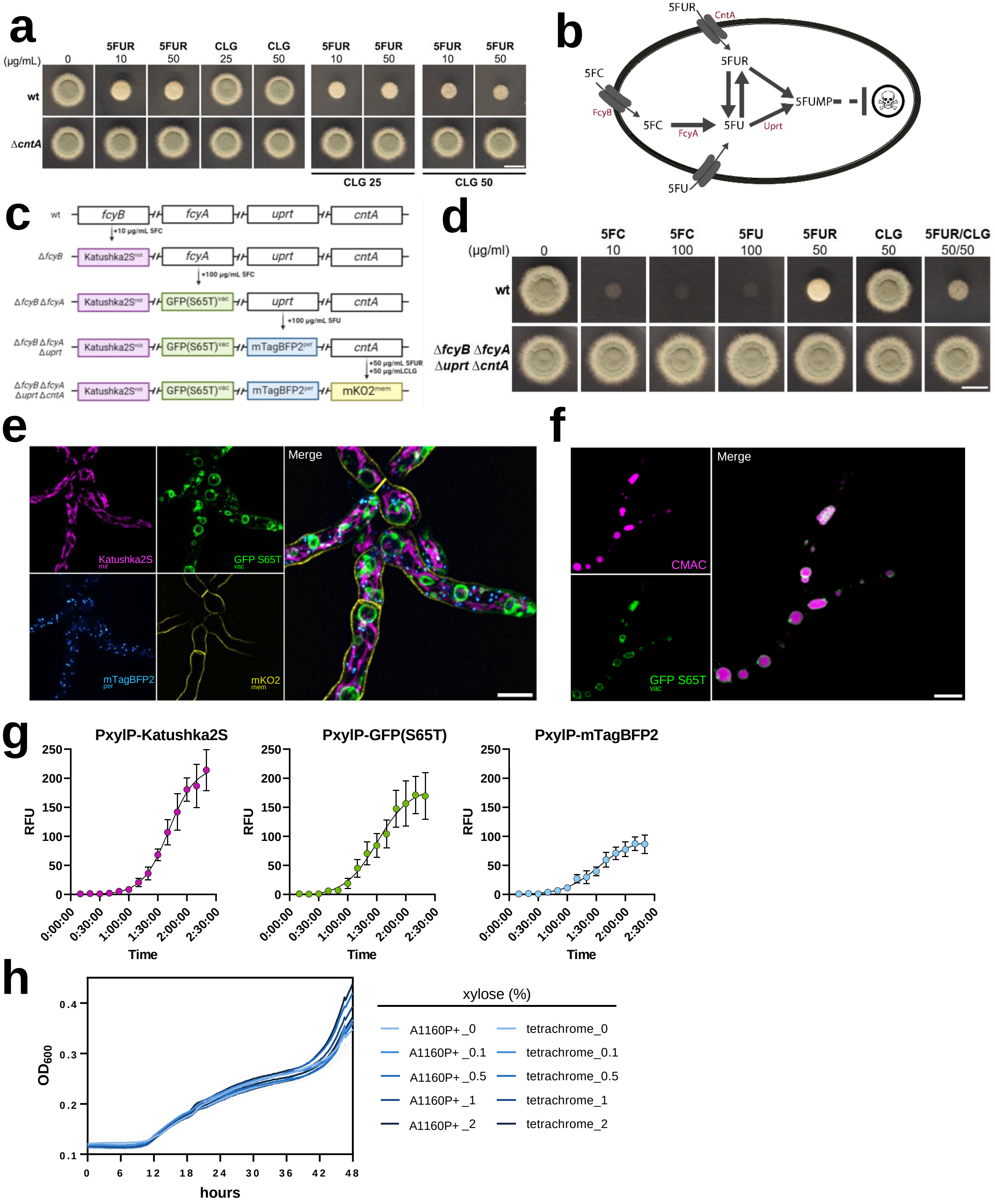

Figure S3. Conditional, xylose-inducible expression of distinct fluorophores. (a) Plate growth-based susceptibility testing of WT and ΔcntA exposed to 5FUR and CLG. The strains were point inoculated on solid AMM pH 5 and incubated for 48 h at 37 °C. Scale bar: 1 cm. (b) Scheme illustrating A. fumigatus proteins involved in both the uptake and metabolisation of 5-fluoropyrimidines through the pyrimidine salvage pathway. (c) The final strategy proposed to perform sequential-counter-selection based on 5-fluoropyrimidine resistance. fcyB, fcyA, uprt and cntA genes are replaced with sequences encoding fluorescent-labelled subcellular markers. (d) Plate growth-based susceptibility testing of WT and the quadruple mutant. strains were point-inoculated on solid AMM pH 5 and incubated for 48 h. Scale bar, 1 cm. (e) Multicolour imaging of the tetrachrome strain. Scale bar: 5 µm. (f) Determination of the subcellular localization of GFP S65T-Vam3 in Aspergillus fumigatus. GFP S65T-Vam3 surrounds with CMAC-labelled vacuoles. Scale bar: 5 µm. (g) PxylP-driven expression of GFP S65T, mTagBFP2, and Katushka2S. A. fumigatus germlings were grown in RPMI-1640 for 18 hours at 30 °C and 1 hour at 37 °C. FP expression was induced by supplementing the medium with 1% xylose and cells were imaged in 10-minute intervals for 2.5 hours via confocal microscopy at 37 °C. FP brightness (RFU) was assessed via ImageJ for 10 germlings per strain. (h) Growth rates were calculated as the slope of growth between 12-18 hours. These were compared by a one-way ANOVA ($F(9, 20)=0.7108$, ns) ($n=3$).

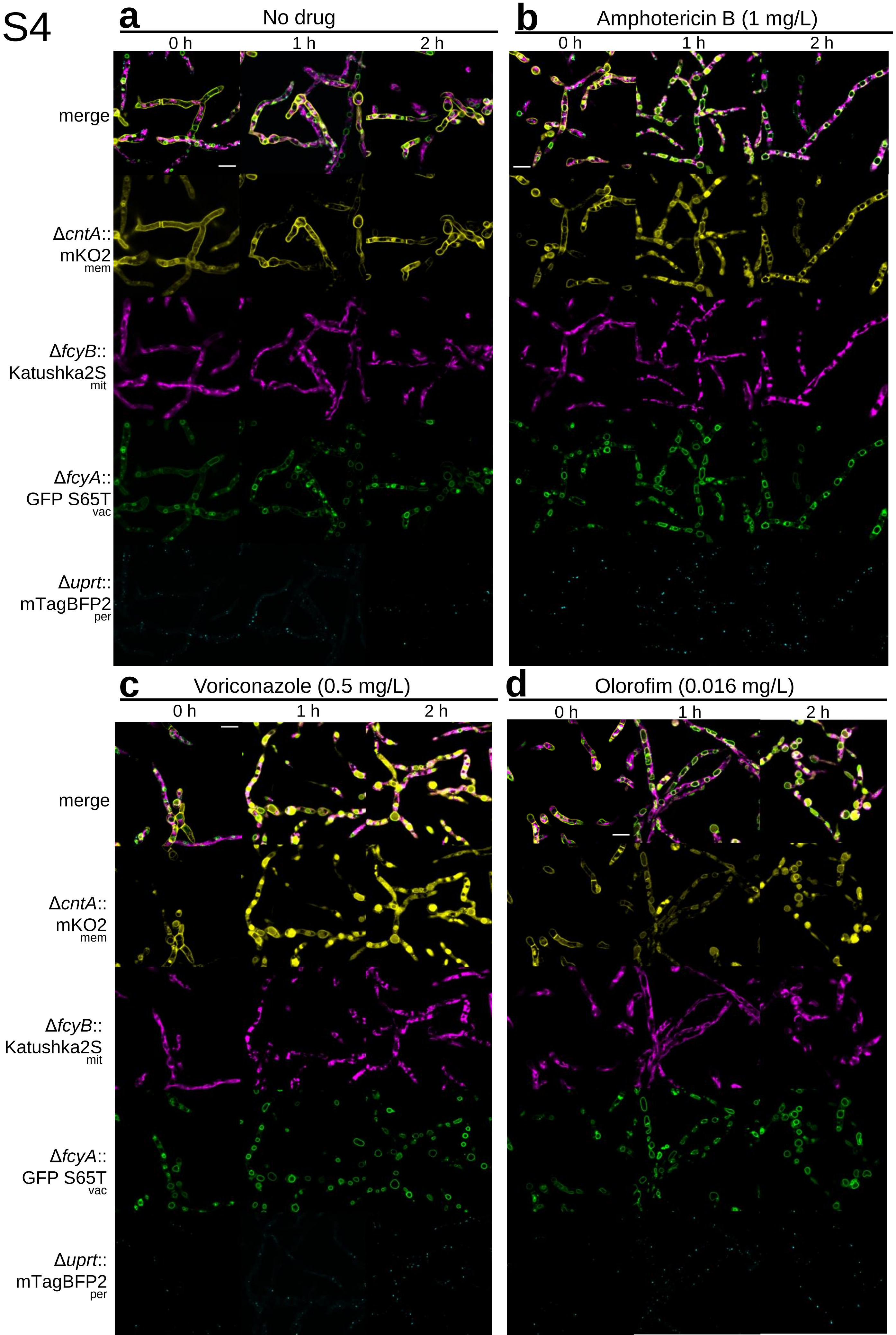
S4
a
No drug
b
Amphotericin B (1 mg/L)
c
Voriconazole (0.5 mg/L)
d
Olorofim (0.016 mg/L)
0 h
1 h
2 h
merge
ΔcntA::
mKO2
mem
ΔfcyB::
Katushka2S
mit
ΔfcyA::
GFP S65T
vac
Δuprt::
mTagBFP2
per

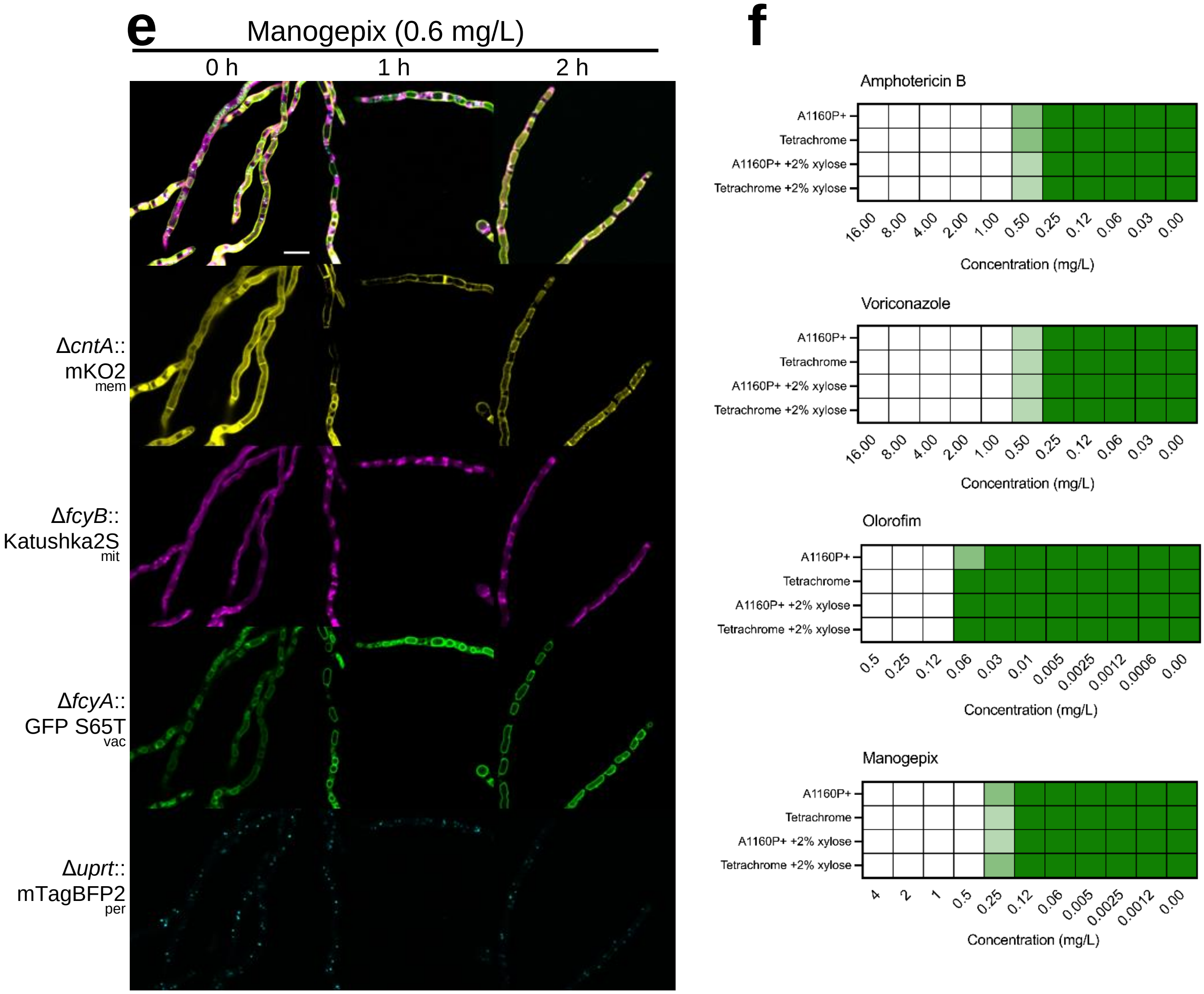

Figure S4. Individual channels for hyphae exposed to (a) no drug, amphotericin B (1 mg/L), (c) voriconazole (0.5 mg/L), (d) olorofim (0.016 mg/L), and (e) manogepix (0.6 mg/L). Scale bar, 10 µm. (f) The tetrachrome strain exhibits the same MIC to amphotericin B, voriconazole, and olorofom, and the same MEC to manogepix, as the parental WT strain (A1160P+). The MIC and MEC values do not change with the addition of xylose. Determination of the MIC and MEC were carried out according to EUCAST guidelines (Guinea et al., 2022).

S5

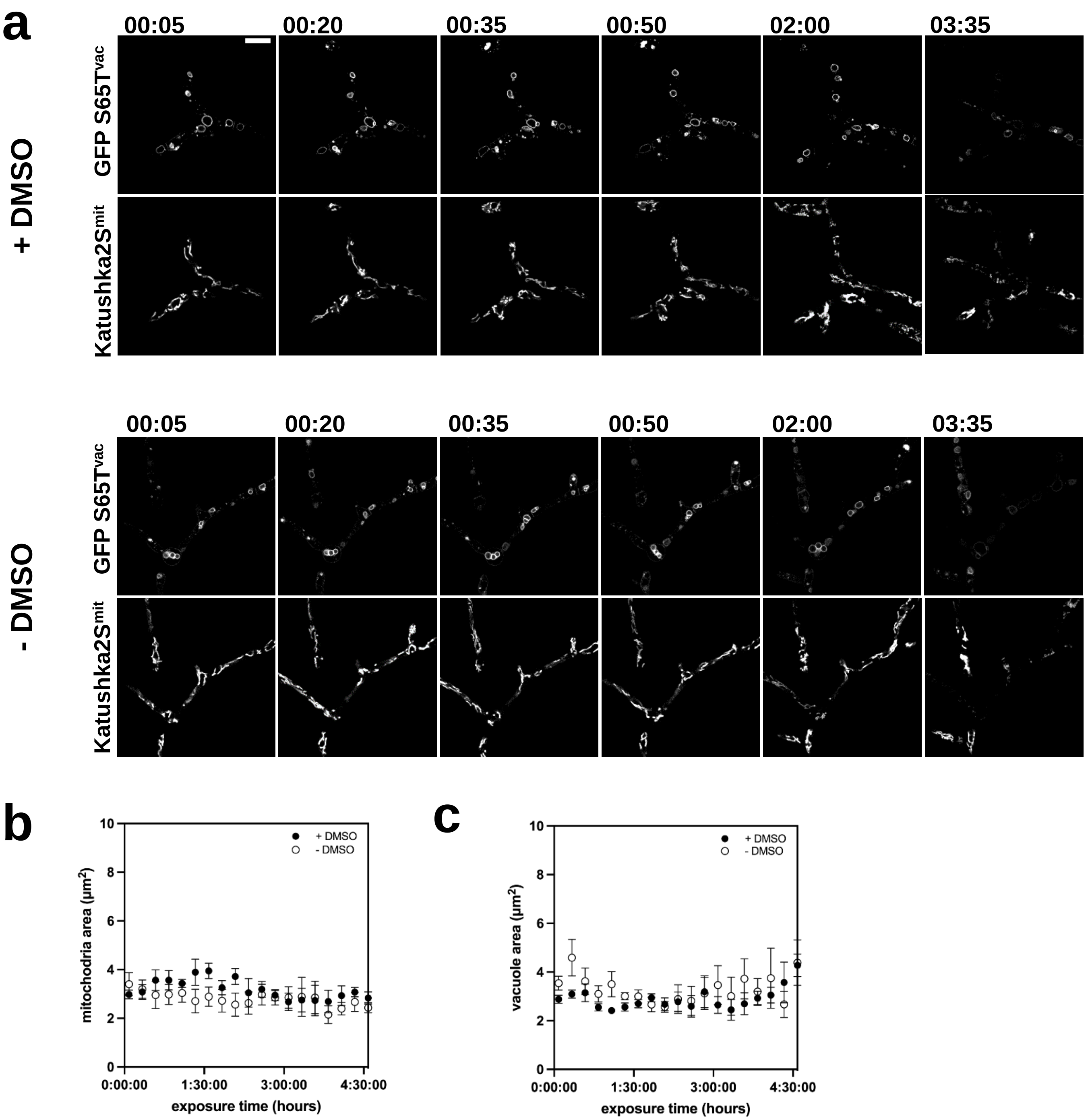

Figure S5. (a) Mitochondria and vacuoles in the tetrachrome strain with and without the addition of 0.01% DMSO. Scale bar, 10 µm. (b) There was no significant effect of time ($t(3.42) = -2.003$, $p = 0.1275$), DMSO ($t(2.45) = -0.831$, $p = 0.479$) or an interaction between the two ($t(178.9) = 0.26$, $p = 0.7953$) on the mitochondria fragment area. (c) There was no significant effect of time ($t(23.16) = 1.679$, $p = 0.10660$), DMSO ($t(8.42) = 1.194$, $p = 0.26491$) or an interaction between the two ($t(180.6) = -1.403$, $p = 0.16227$) on the vacuole area.
