# Supplementary material for: Shining a light on the impact of antifungals on Aspergillus fumigatus subcellular dynamics through fluorescence imaging
Source: Antimicrob Agents Chemother. 2024 Oct 15;68(11):e00803-24. doi: 10.1128/aac.00803-24 (PMC11539212; doi:10.1128/aac.00803-24)
Supplement: Supplemental methods — Supplemental materials and methods on strain generation. [file aac.00803-24-s0002.docx]

**Supplementary materials and methods**

1. *Generation of plasmids and strains expressing fluorescent proteins in the cytoplasm*

Plasmids carrying sequences encoding GFP S65T, mKO2, mTagBFP2, and Katushka2S under the control of *A. nidulans* *PgpdA* were constructed as follows. A common backbone was amplified using the plasmid pAN7-1 as a DNA template for the primer set BBgpdA-FW/RV. While the insert coding for GFP S65T was amplified from pgfpcccA (Gsaller et al., 2012), the inserts for mKO2, mTagBFP2 and Katushka2S were amplified from synthetic gene strands (Integrated DNA Technologies, Coralville, IA, USA; codon adapted *for A. fumigatus*), with the primer pairs PgpdAsGFP-FW/RV, PgpdAmKO2-FW/RV, PgpdAmTagBFP2-FW/RV and PgpdAK2S-FW/RV, respectively, each containing 20 bp overlapping ends to the backbone. The backbones were subsequently assembled with each amplified insert using NEBuilder® yielding plasmids pFG36, pBM12, pBM13 and pFG39, which were used as DNA templates for the primer set hph-FW/RV to generate the expression cassettes under the control of the *A. nidulans gpdA* promoter (*PgpdA*) and *trpC* terminator (*TtrpC*).

To generate integrative constructs comprising inducible GFP S65T, mTagBFP2 and Katushka2S expression cassettes, first a backbone containing *Penicillium chrysogenum PxylP* and *Aspergillus terreus TtrpC* was amplified from pΔfcyB_mKate2xyl (Birštonas et al., 2020). Next, the coding sequences with 20 bp overlapping ends to the backbone were amplified from pFG36, pBM13 and pFG39 using primer pairs PxylPsGFP.2-FW/RV, PxylPmTagBFP2-FW/RV, PxylPK2S-FW/RV and assembled with the backbone using NEBuilder®, yielding plasmids pESV12, pPM29, and pESV18 were *Not*I-linearised and transformed in the CEA10 Δ*ku80* derivative A1160P+ (here termed WT), which served as parental strain in this work.

1. ∆*cntA::ble* generation

The sequence encoding CntA (AFUB_001570) was replaced by the phleomycin resistance cassette *ble*. Therefore, deletion constructs comprising approximately 1 kb of *cntA*5´ and 3´ nontranslated regions (NTRs) linked to the central antibiotic resistance cassette were generated using fusion PCR as previously described (Fraczek et al., 2013). Correct integration of the transformed construct was confirmed by Southern blot analysis.

1. *Construction of cassettes to generate the tetrachrome strain*

To obtain DNA templates targeting specific loci (*fcyB, fcyA, uprt* and/or *cntA*) required to perform counter-selection-based fungal transformation, several constructs were made. Generally, the poly-glycine-serine (G4S) linker (Klein et al., 2014) was used to improve the flexibility during protein tagging. Initially, the plasmid pESV13 was obtained by linearizing the pfcyB (Birštonas et al., 2020) with the primer set BBdel-FW/RV and fusing it with a *PxylP*-expression cassette encoding the green fluorescent protein variant GFP S65T, containing N-terminal mitochondrial targeting sequence (CitA_40_::GFP S65T derived from citrate synthaseGFP S65T (Min et al., 2010), amplified from pX-sGFPMit (Birštonas et al., 2020) using the pX-cass-FW/RV primers. pESV13 served as a DNA template for the primers pX-FW.2 and ∆fcyBPxMitBB-RV to generate a backbone carrying a PxylP-citA_40_ cassette directed to the *fcyB* locus. Subsequently, a fragment encoding Katushka2S was amplified from a synthetic gene strand (Integrated DNA Technologies, Coralville, IA, USA; codon adapted for *A. fumigatus*) with the primers citA-K2S-Fw and pX-K2S-RV and fused with the previous backbone, obtaining a plasmid carrying a *PxylP*-driven mitochondrial-Katushka2S reporter directed to the *fcyB* locus, here termed pESV40.

To generate strains encoding a *PxylP*-inducible syntaxin Vam3 tagged with GFP S65T, the plasmid pESV39 to label vacuolar membranes was generated as follows. BlastP analyses were performed to determine the ortholog of *A. oryzae* AoVam3 (Shoji et al., 2006) in *A. fumigatus*, then the Vam3 (AFUB_064200) and GFP S65T coding sequences were amplified from pFG36 genomic DNA and the plasmid pgfpcccA using the primers sGFP-vam3-FW1/RV1 and sGFP-vam3-FW2/RV2, respectively. Both fragments were fused with a backbone obtained from the template plasmid p∆fcyA_cyp51APxylP (Baldin et al., 2022) with the primers pX-FW.2 and pX-RV.2, yielding pESV39, which allowed integration of the construct into the *fcyA* locus.

The plasmid pESV9 was generated after fusing a backbone containing the *xylP* promoter with the coding sequence of the blue fluorescent protein mTagBFP2 carrying a C-terminal peroxisomal targeting sequence 1, PTS1 (SKL tripeptide) (Olivier & Krisans, 2000), both PCR-generated from the pX-lacZ and the pANmTagBFP2 (Birštonas et al., 2020) using the primers pX-FW.2/RV.2 and mTagBFPSKL- FW/RV respectively. This respective plasmid was used as the template for the primer set pX-cass-FW/RV to generate a xylose inducible mTagBFP2-SKL cassette. In parallel, a backbone containing approximately 1 kb of 5´and 3´NTRs of the *uprt* locus was amplified from the plasmid p∆uprt (Baldin et al., 2022) using the primers BBdel-FW/RV. This backbone was fused with the *PxylP*-inducible mTagBFP2-SKL cassette, generating pESV26.

To generate the required backbone for the construction of plasmids targeting the *cntA* locus, the plasmid pESV33 was assembled as previously described for pfcyB, p∆fcyA_cyp51APxylP and p∆uprt constructions. Briefly, a pUC19 plasmid was linearized with the primer set pUC19L-FW/RV and fused with approximately 1-kb 5´and 3´NTRs of *cntA*, amplified from genomic DNA with the primers cntAN1.2-FW/cntA2.2-RV and cntA3.2-FW/cntAN2.2-RV, respectively. pESV33 was used to generate a plasmid carrying a *PxylP*-inducible cassette encoding UapC, a major purine transporter localized in the cell membrane (Valdez-Taubas et al., 2000), tagged with the fluorescent protein mKO2. The fragments required for this construction, named pESV24, were amplified from the plasmid pX-lacZ, genomic DNA (*uapC*, AFUB_091490) and a synthetic *mKO2* gene strand (Integrated DNA Technologies, Coralville, IA, USA; codon adapted for *A. fumigatus*), using the primers pX-FW.2/RV.2, uapCmKO2-FW/RV and mKO2uapC-FW/RV, respectively. At last, the pESV33 was linearized with the primer set BBdel-FW/RV and fused with the *PxylP*- inducible *uapC*-*mKO2* cassette, amplified from pESV24 with the primers pX-cass-FW/RV, giving rise to pESV36, a plasmid targeting the *cntA* locus, required for the generation of strains expressing fluorescent signal at the cell membrane.

1. *Southern blots*

2.5 μg of DNA extracted from conidia was digested using *Xho*I (NEB) following the manufacturer’s instructions for 18 hours at 37 °C. DNA was separated by gel electrophoresis. The gel was washed in water for 5 minutes with shaking at 30 rpm. The gel was denatured in denaturing buffer (1.5 M NaCl, 0.5 M NaOH) for 30 minutes with shaking at 30 rpm, washed with water, and neutralised in neutralisation buffer (1.5 M NaCl, 0.5 M Tris HCl, pH 7.5) for 20 minutes with shaking. DNA was transferred to an Amersham Hybond-NX nylon membrane (GE Healthcare) using transfer buffer (150 mM NaCl, 15 mM sodium citrate, pH 7) by capillary action for 18 hours. The membrane was crosslinked using a UV Stratalinker 1800 (Stratagene) and subsequently pre-hybridised in 10 X Saline Sodium Citrate (SSC) buffer (0.5 M NaCl, 5% (w/v) blocking agent) for one hour at 46.4 °C. The probe was generated via PCR using a PCR DIG Probe Synthesis Kit (Roche) following the manufacturer’s instructions. The probe was designed to be complementary to the *PgdpA* promoter of the fluorescent protein expression cassette. The probe was diluted in DIG Easy Hyb (Roche) to 100 ng/mL and 25 mL of probe solution was added to the membrane after removal of the pre-hybridisation solution SSC buffer. Hybridisation occurred at 46.4 °C for 18 hours. The membrane was washed in 100 mL of 2 X SSC, 0.5% SDS at 68 °C for 15 minutes and then washed in 0.5 X SSC, 0.5% SDS at 68 °C for 15 minutes. The membrane was transferred into a plastic tray and blocked in blocking buffer (100 mM maleic acid, 150 mM NaCl, 1% (w/v) blocking agent, pH 7.5) for two hours at room temperature with shaking at 30 rpm and then discarded. Anti-Digoxigenin-AP Fab fragments (Roche) were diluted to 75 mU/mL in blocking buffer and the solution was added to the membrane and incubated for 30 minutes at room temperature with shaking at 30 rpm. The antibody solution was discarded and the membrane was washed 3 times each for 15 minutes in washing buffer (100 mM maleic acid, 150 mM NaCl, 0.5% Tween-20, pH 7.5) with shaking at 120 rpm. The membrane was rinsed in detection buffer (100 mM Tris-HCl, 100 mM NaCl, pH 9.5) and Ready to Use CSPD (Roche) was added dropwise to the membrane before imaging on a Gel Doc XRS System (Bio-rad).
